# Serum fatty acids, biochemical indices and antioxidant status in goats fed canola oil and palm oil blend

**DOI:** 10.1186/s40781-016-0088-2

**Published:** 2016-02-08

**Authors:** Kazeem D. Adeyemi, Azad B. Sabow, Zeiad A. Aghwan, Mahdi Ebrahimi, Anjas A. Samsudin, Abdul R. Alimon, Awis Q. Sazili

**Affiliations:** Department of Animal Science, Faculty of Agriculture, Universiti Putra Malaysia, Serdang, Selangor 43400 Malaysia; Halal Products Research Institute, Universiti Putra Malaysia, Serdang, Selangor 43400 Malaysia; Animal Production Laboratory, Institute of Tropical Agriculture, Universiti Putra Malaysia, Serdang, Selangor 43400 Malaysia; Department of Veterinary Preclinical Sciences, Faculty of Veterinary Medicine, Universiti Putra Malaysia, Serdang, Selangor 43400 Malaysia; Department of Animal Production, University of Ilorin, Ilorin, PMB 1515 Nigeria; Department of Animal Resource, University of Salahaddin, Erbil, Kurdistan Region Iraq; Department of Animal Science, College of Agriculture, University of Mosul, Mosul, Iraq

**Keywords:** Carotenoid, Catalase, Cholesterol, Glutathione peroxidase, Superoxide dismutase, Tocopherol

## Abstract

**Background:**

Dietary supplementation of unsaturated fats in ruminants, if not stabilized, can instigate oxidative stress which can have negative impact on production performance and enhance the susceptibility to various diseases. The current study examined the effect of dietary 80 % canola oil and 20 % palm oil blend (CPOB) on serum fatty acids, antioxidant profile and biochemical indices in goats. Thirty Boer bucks (4–5 months old; initial BW, 20.34 ± 0.77 kg) were randomly assigned to diets containing 0, 4 or 8 % CPOB and fed daily for a period of 90 days. Blood was sampled from the goats on 0, 30, 60 and 90 days of the trial and the serum was analyzed for fatty acids, cholesterol, glucose, total protein, antioxidants and lipid oxidation.

**Results:**

Neither diet nor sampling time influenced serum TBARS value, catalase, glutathione peroxidase and superoxide dismutase activities, LDL cholesterol, VLDL cholesterol, triglycerides, glucose and total protein. Goats fed 4 and 8 % CPOB had higher (*P* < 0.05) total cholesterol and HDL cholesterol than the control goats on day 30, 60 and 90. The proportion of C15:0 decreased with increasing level of CPOB on day 30 and 60. Serum C18:1n-9 increased with increasing level of CPOB in diet on day 60. The proportion of C18:3n-3 and C22:5n-3 increased (*P* < 0.05), while the proportion of C18:2n-6 decreased (*P* < 0.05) with increase in the level of CPOB on day 60 and 90. Dietary CPOB did not affect serum total carotenoid and δ-tocopherol but did increase (*P* < 0.05) α and γ-tocopherol.

**Conclusion:**

Dietary canola oil and palm oil blend could be supplemented in diets without instigating oxidative stress in goats.

## Background

The utilization of dietary fats in ruminant nutrition is a continued research endeavor. Due to the high energy density and being low priced, dietary fats can be used to solve the glitches of energy supply in ruminants [1–3]. Also, dietary unsaturated fats can be utilized to alter the fatty acid (FA) profile of ruminant meat [2] and milk [3]. However, dietary supplementation of unsaturated fats especially polyunsaturated fatty acids (PUFA), if not stabilized, could instigate oxidative stress in animals [3, 4]. Oxidative stress could alter physiological functions, impart negatively on growth performance and enhance susceptibility to various diseases [3–6].

The level and type of dietary fat influence the biochemical parameters of the blood, which are sensitive indicators of the state of health and reflect the intensity of metabolic processes taking place in the animals [3–5]. It is commonly assumed that animals exposed to oxidative stress respond with compensatory induction of antioxidant enzymes [4–7]. Nonetheless, the effect of dietary antioxidants and fats on the activities of antioxidant enzymes is contentious [4, 6, 7]. In addition, the impact of dietary fat on serum biochemical indices in ruminants has been highly variable and inconsistent [1, 4] in the published literature. This scenario justifies the need for additional studies in different production systems to permit tailored decisions and informed choices in the utilization of fat supplements in ruminant nutrition.

Albeit, dietary supplementation of vitamin E provides a practicable alternative to prevent oxidative stress in animals fed unsaturated fats, this practice can impose an extra financial burden on livestock farmers. Some vegetable oils are rich in natural antioxidants [8]. Thus, the utilization of such oils in animal nutrition may be an effective and economical method of attenuating dietary fat-induced oxidative stress in animals [8, 9]. One of such oils is canola oil which is an excellent source of polyphenols, phytosterol and tocopherol [10]. Palm oil is rich in lipid soluble antioxidants such as tocopherol, tocotrienols, lycopenes, ubiquitone and carotenoids [11]. Given the attributes of both canola and palm oils, it was proposed that their mix could be utilized in ruminant nutrition without deleterious effects on serum antioxidant status and biochemical indices. Companion in vitro [12] and in vivo [13] studies have shown that dietary blend of 80 % canola oil and 20 % palm oil did not have deleterious effects on rumen fermentation, nutrient intake and digestibility and growth performance in goats. Thus, the current study aimed at elucidating the consequences of utilizing 80 % canola oil and 20 % palm oil blend (CPOB) in ruminant nutrition on the serum antioxidant status, fatty acids and biochemical indices in goats.

## Methods

### Animal welfare and ethics

The study was conducted according to the guidelines approved by the Universiti Putra Malaysia Institutional Animal Care and Use Committee. The health and welfare of the goats were monitored by a qualified veterinarian who is a member of the research team.

### Experimental animals and diet

Thirty Boer bucks of 4–5 months old, having initial body weight of 20.34 ± 0.77 kg were used for the trial. The animals were treated against endo and ecto parasites prior the commencement of the trial. Each animal was individually housed in wooden slatted floor pen (1.20 m × 0.80 m × 0.70 m) furnished with drinking and feeding facilities. The goats were randomly assigned to diets containing on DM basis 0, 4 or 8 % CPOB fed for 90 days following 2 weeks of adaptation. The diets were formulated to meet the nutritional requirements of growing goats following the recommendation of NRC [14]. Animals were fed twice a day at 8.30 and 14.30 with free access to clean water. The chemical and fatty composition and antioxidant profile of the diets are presented in Table 1.

**Table 1:** Chemical, fatty acid and antioxidant composition of dietary treatments

	Levels of CPOB^a^ (% )			SE	*P value*
Chemical composition, g/kg DM	0	4	8		
Dry matter	676.96	678.99	680.73	32.00	0.56
Crude Protein	142.72	143.73	143.92	14.23	0.11
Ether extract	23.00	63.50	111.10	2.19	0.01
Ash	68.40	65.80	62.60	4.11	0.17
Organic matter	931.60	934.20	935.50	44.22	0.65
Nitrogen free extract	165.56	139.67	124.51	15.06	0.17
Acid detergent fibre	350.40	332.80	325.20	18.99	0.26
Neutral detergent fibre	635.24	626.72	620.60	27.12	0.80
Metabolizable energy, MJ/Kg DM	11.59	11.61	11.62	0.78	0.10
Ca %	1.02	1.05	1.04	0.02	0.18
P %	0.52	0.54	0.54	0.01	0.12
Fatty acid (g/100 g total FA)					
C12:0	0.07	0.07	0.08	0.01	0.45
C14:0	3.35	1.38	0.99	0.05	0.04
C16:0	17.64	16.14	14.90	1.89	0.14
C16:1	0.52	0.31	0.29	0.01	0.13
C18:0	3.52	3.00	2.73	0.02	0.23
C18:1n-9	24.17	40.06	50.37	4.19	0.01
C18:2n-6	44.57	32.00	23.03	3.20	0.02
C18:3n-3	6.70	7.04	7.90	0.12	0.15
∑SFA	24.52	21.00	18.70	1.15	0.36
∑UFA	75.48	79.00	81.30	8.23	0.03
n-6:n-3	6.66	4.54	2.92	0.66	0.01
Total FA (g/kg DM)	15.83	37.09	52.27	2.03	0.01
Antioxidants					
Total carotenoid (mg/kg)	14.81	16.71	19.86	1.92	0.02
α-tocopherol (mg/kg)	101.12	112.47	123.21	7.81	0.01
γ-tocopherol (mg/kg)	10.22	34.55	49.17	4.50	0.01
δ-tocopherol (mg/kg)	1.21	3.45	5.93	0.04	0.02

^a^80 % canola oil and 20 % palm oil blend. ∑SFA = (C12:0 + C14:0 + C16:0 + C18:0), ∑MUFA = (C16:1+ C18:1), ∑PUFA = (C18:2n-6 + C18:3n-3) n-6:n-3 = (C18:2n-6÷C18:3n-3)

### Blood sampling

Blood samples were collected through jugular venipuncture into plain serum bottles on 0, 30, 60 and 90 days of the experiment. The blood samples were centrifuged at 4000 g for 15 min and the resulting supernatant was collected into centrifuged tubes and stored at −80 °C until further analysis.

### Determination of serum cholesterol, glucose and total protein

The serum total cholesterol, high density lipoprotein (HDL) cholesterol, glucose, triglycerides and total protein was determined using automatic analyzer (Automatic analyzer 902, Hitachi, Germany). The low density lipoprotein (LDL) cholesterol was estimated using the equation of Friedwald et al. [15]: LDL cholesterol = Total cholesterol - HDL cholesterol- very low density lipoprotein (VLDL) cholesterol. Where VLDL cholesterol = Triglycerides/5

### Determination of total carotenoid

The carotenoid contents in feed and serum samples was extracted and determined following the method described by Adeyemi et al. [16]. Two gram of each sample was homogenized with 10 mL acetone. The contents were stirred for 30 min and 10 mL of acetone was used to rinse the flask and to re-extract the residue. Thereafter, extracts were pooled and 1 mL of distilled water was added to the extract. A 5 mL n-hexane was added to the mixture and centrifuged at 3500 g for 15 min. The absorbance of the hexane layer was read at 450 nm using a spectrophotometer (Secomam, Domont, France). Total carotenoid content was estimated by the following formula:

$$ \mathrm{Conc}.\ \left(\upmu \mathrm{g}/\mathrm{g}\right)=\left({\mathrm{A}\times \mathrm{V}\times 10}^4\right)/\left(2592\times \mathrm{W}\right) $$

Where A: absorbance
V: Volume of n-hexane (mL)
W: Sample weight

### Determination of tocopherol

Extraction of tocopherol from serum and feed samples followed the method of Kamal-eldin et al. [17]. Quantification of tocopherol contents was done with Agilent 1200 series HPLC as described by Pegg and Amarowic [18]. The column used was C_30_ YMC^TM^ carotenoid (250 mm x 4.6 mm. i.d, 5 μm) (YMC, USA). An isocratic mobile phase made up of 99 % n-hexane and 1 % Isopropanol was used. The flow rate and injection volume was 0.5 mL/min and 20 μL respectively. The UV detection was monitored at 295 nm. The isomers of tocopherol were quantified by comparing the peak area of sample with those of tocopherol standards in the HPLC controller software.

### Determination of fatty acids

The fatty acids (FA) in the feed and serum samples were extracted in chloroform: methanol (2:1, v/v) as described by Rajion et al. [19]. The FAs were transmethylated into their fatty acid methyl esters (FAME) using 0.66 N KOH in methanol and 14 % methanol boron trifluoride (BF_3_) in accordance to the method of AOAC [20]. Heneicosanoic acid was used as internal standard. The FAME was separated in a gas chromatograph (Model 6890 Agilent Technologies, USA). The column used was fused silica capillary (Supelco SP-2330, 30 m, 0.25 mm ID, 0.20 μm film thickness). The carrier gas was high purity nitrogen at 40 mL/min. High purity nitrogen and compressed air were used for the flame ionization detector in the chromatograph. The oven temperature was set at 100 °C, for 2 min and warmed to 170 °C at 10 °C/min, held for 2 min, warmed to 230 °C at 5 °C/min, and then held for 20 min to facilitate optimal separation. The FA was identified by comparing the sample relative FAME peak and retention times with that of fatty acid methyl standards (Sigma chemical).

### Lipid oxidation

Lipid oxidation was measured as 2-thiobarbituric acid reactive substances (TBARS) using QuantiChrom™ TBARS Assay Kit (DTBA-100, BioAssay Systems, USA) following the manufacturer’s procedure.

### Antioxidant enzyme activities

Glutathione peroxidase (GPx) was measured with the aid of EnzyChromTM Glutathione Peroxidase Assay Kit EGPX-100, (BioAssay Systems, USA), the Superoxide Dismutase (SOD) activity was measured with the aid of Cayman SOD Assay kit 706002, (Cayman chemical) while the catalase activity was measured using Cayman Catalase Assay Kit 707002, (Cayman chemical) following the manufacturer’s procedure.

### Statistical analysis

The data obtained for all serum parameters were subjected to a repeated measure analysis of variance using the mixed procedure of SAS [21] in which dietary treatment, days of sampling and interaction between dietary treatments and sampling days were fitted as fixed effects while goats and baseline values of parameters were fitted as random effects. Tukey HSD test was used to separate means at *P* < 0.05 significance level.

## Results

### Chemical and fatty acid composition of diets

The chemical, fatty acid, and antioxidant composition of the dietary treatments is shown in Table 1. The forage portion of the diet was oil palm frond (OPF) which accounted for 50 % of the basal diet in all treatments. The oil blend was incorporated into the concentrate portion of the diets which consisted of 22 % corn grain, 17 % soybean meal, 7.5 % palm kernel cake and 2 % rice bran and 0.5 % limestone, 0.5 % salt and 0.5 % mineral vitamin premix. The concentrate portion was adjusted to make the diet isocaloric and isonitrogenous. Dietary DM, NDF, ADF, crude protein and energy were similar across the treatments. However, dietary ether extract increased (*P* < 0.05) in response to incremental level of CPOB in diet. Addition of CPOB increased the concentration of total fatty acids, C18:3n-3 and C18:1n-9 but reduced the concentration of C18:2n-6 and C14:0 and the n-6/n-3.

### Serum cholesterol, glucose and total protein

The serum biochemical parameters of goats fed graded levels of CPOB are shown in Table 2. Diet and sampling day did not affect the concentration of serum glucose, VLDL cholesterol, LDL cholesterol, triglycerides and total protein. Goats fed 4 and 8 % CPOB had higher (*P* < 0.05) total cholesterol and HDL cholesterol than the control goats on day 30, 60 and 90. There was a diet x sampling day interaction (*P* < 0.05) for total cholesterol and HDL cholesterol. Serum total cholesterol and HDL cholesterol increased (*P* < 0.05) in oil-fed goats as sampling day progressed.

**Table 2:** Effect of diet and sampling time on serum biochemical parameters (mean ± standard error) in goats

		Level of CPOB^g^			*P* value	
Parameter (mmol/L)	Sampling day	0	4	8	Diet	Diet x sampling
Total cholesterol	0	2.51 ± 0.3	2.05 ± 0.3^d^	2.28 ± 0.5^d^	0.20	
	30	1.85 ± 0.1^b^	2.09 ± 0.3^abd^	2.55 ± 0.4^ad^	0.05	0.03
	60	2.54 ± 0.5^b^	3.75 ± 0.4^ae^	3.27 ± 0.4^ae^	0.02	
	90	2.09 ± 0.2^b^	2.58 ± 0.3^ad^	2.44 ± 0.2^ad^	0.04	
	*P* value	0.54	0.04	0.02		
HDL cholesterol	0	1.57 ± 0.1	1.34 ± 0.3	1.33 ± 0.3	0.27	
	30	0.94 ± 0.2^c^	1.35 ± 0.3^b^	1.66 ± 0.4^a^	0.02	
	60	1.64 ± 0.3^b^	2.30 ± 0.8^a^	2.08 ± 0.3^a^	0.01	0.01
	90	1.09 ± 0.3^b^	1.49 ± 0.3^a^	1.42 ± 0.2^a^	0.02	
	*P* value	0.21	0.23	0.12		
LDL cholesterol	0	0.94 ± 0.2	0.74 ± 0.1	0.95 ± 0.2	0.37	
	30	0.91 ± 0.1	0.84 ± 0.3	0.89 ± 0.1	0.07	
	60	0.90 ± 0.2	1.45 ± 0.2	1.20 ± 0.3	0.21	0.23
	90	1.00 ± 0.3	1.07 ± 0.3	1.02 ± 0.1	0.95	
	*P* value	0.15	0.12	0.08		
VLDL cholesterol	0	0.08 ± 0.0	0.09 ± 0.0	0.10 ± 0.0	0.77	
	30	0.04 ± 0.0	0.06 ± 0.0	0.06 ± 0.0	0.14	
	60	0.09 ± 0.0	0.13 ± 0.0	0.10 ± 0.0	0.24	0.56
	90	0.04 ± 0.0	0.05 ± 0.0	0.05 ± 0.0	0.16	
	*P* value	0.41	0.07	0.06		
Triglycerides	0	0.43 ± 0.0	0.44 ± 0.1	0.50 ± 0.1	0.76	
	30	0.48 ± 0.1	0.65 ± 0.1	0.51 ± 0.1	0.11	
	60	0.20 ± 0.0	0.32 ± 0.0	0.29 ± 0.1	0.31	0.84
	90	0.21 ± 0.0	0.27 ± 0.0	0.21 ± 0.0	0.26	
	*P* value	0.09	0.21	0.33		
Glucose	0	2.50 ± 0.1	2.34 ± 0.2	2.80 ± 0.2	0.19	
	30	3.18 ± 0.2	2.84 ± 0.2	3.02 ± 0.2	0.57	
	60	2.31 ± 0.3	2.38 ± 0.4	2.50 ± 0.2	0.93	0.67
	90	2.83 ± 0.1	2.62 ± 0.3	2.81 ± 0.3	0.85	
	*P* value	0.11	0.21	0.36		
Protein (g/L)	0	72.90 ± 4.4	75.66 ± 3.7	76.55 ± 1.9	0.75	
	30	65.80 ± 4.5	68.10 ± 3.8	71.88 ± 4.1	0.17	
	60	72.25 ± 4.3	80.12 ± 4.2	81.07 ± 3.5	0.07	0.35
	90	64.80 ± 2.2	67.52 ± 2.7	63.65 ± 2.4	0.54	
	*P* value	0.57	0.22	0.45		

^a, b, c^means having different superscript along the same row are significantly different (*P* < 0.05). ^d, e^means having different superscript along the same column are significantly different (*P* < 0.05). ^g^80 % canola oil and 20 % palm oil blend

### Serum fatty acids

The serum fatty acid profile of goats fed varying level of CPOB is shown in Tables 3 and 4. Neither diet nor sampling day influenced the proportion of C14:0, C16:0, C16:1n-7 and C18:0 (Table 3), C18:3n-6; C20:4n-6, C20:5n-3, C22:6n-3 and total FA (Table 4). However, goats fed 4 and 8 % CPOB had higher proportion (*P* < 0.05) of C18:3n-3 (on day 30, 60 and 90), C22:5n-3 (on day 60 and 90) and lower (*P* < 0.05) proportion of C15:0 (on day 30 and 60) and C18:2n-6 (on day 60 and 90) compared with the control goats. Increasing sampling time decreased (*P* < 0.05) the proportion of C15:0 in goats fed 4 and 8 % CPOB. On day 60, increasing level of CPOB enhanced (*P* < 0.05) the proportion of C18:1n-9.

**Table 3:** Effects of diet and sampling time on saturated and monounsaturated fatty acids (mean ± standard error) in serum of goats

		Levels of CPOB^g^			*P* value	
Fatty acid (% of total fatty acid)	Sampling day	0	4	8	Diet	Diet x sampling day
C14:0	0	3.68 ± 0.4	3.83 ± 0.2	3.71 ± 0.3	0.82	0.20
	30	4.32 ± 0.9	3.08 ± 0.5	3.45 ± 0.6	0.50	
	60	3.40 ± 0.6	3.07 ± 0.6	3.09 ± 0.6	0.52	
	90	2.94 ± 0.3	2.05 ± 0.3	2.56 ± 0.3	0.58	
	*P* value	0.39	0.12	0.60		
C15:0	0	3.03 ± 0.2	3.16 ± 1.1^d^	3.31 ± 0.2^d^	0.18	0.01
	30	1.91 ± 0.3^a^	0.89 ± 0.1^be^	0.79 ± 0.1^be^	0.01	
	60	2.98 ± 0.2^a^	1.04 ± 0.4^be^	1.75 ± 0.3^be^	0.04	
	90	1.99 ± 0.1^a^	1.61 ± 0.3^ae^	2.03 ± .92^ae^	0.35	
	*P* value	0.30	0.01	0.02		
C16:0	0	18.46 ± 0.7	18.28 ± 1.1	18.94 ± 1.0	0.51	0.67
	30	17.66 ± 0.5	19.36 ± 2.7	17.33 ± 1.1	0.77	
	60	18.40 ± 2.0	18.64 ± 1.2	18.53 ± 1.4	0.62	
	90	17.23 ± 0.6	18.66 ± 2.9	18.89 ± 1.7	0.19	
	*P* value	0.90	0.23	0.56		
C16:1n-7	0	3.23 ± 0.2	3.64 ± 0.2	3.95 ± 0.1	0.53	0.91
	30	2.82 ± 0.3	2.66 ± 0.5	3.38 ± 0.6	0.67	
	60	2.12 ± 2.0	2.58 ± 0.3	3.26 ± 1.3	0.08	
	90	2.83 ± 0.3	2.87 ± 1.5	2.82 ± 0.2	0.27	
	*P* value	0.43	0.11	0.22		
C18:0	0	14.76 ± 1.1	14.91 ± 1.1	16.66 ± 1.2	0.47	0.22
	30	18.38 ± 1.1	19.00 ± 2.0	19.58 ± 1.2	0.79	
	60	18.91 ± 1.1	18.31 ± 2.9	17.06 ± 0.9	0.49	
	90	18.00 ± 1.8	16.41 ± 2.3	16.53 ± 1.3	0.72	
	*P* value	0.36	0.47	0.23		
C18:1n-9	0	25.92 ± 1.3	22.10 ± 1.2	23.07 ± 2.1	0.30	0.23
	30	21.17 ± 0.7	23.02 ± 1.4	21.49 ± 1.3	0.10	
	60	17.58 ± 0.8^a^	19.83 ± 0.8^b^	23.85 ± 1.2^c^	0.01	
	90	20.81 ± 2.6	18.88 ± 1.6	18.00 ± 1.8	0.45	
	*P* value	0.67	0.14	0.33		

^a, b c^means having different superscript along the same row are significantly different (*P* < 0.05). ^d, e, f^means having different superscript along the same column are significantly different (*P* < 0.05). ^g^80 % canola oil and 20 % palm oil blend

**Table 4:** Effect of diet and sampling time on polyunsaturated fatty acids (mean ± standard error) and total fatty acids in serum of goats

		Level of CPOB^g^ (%)			*P* value	
Fatty acid (% of total fatty acid)	Sampling days	0	4	8	Diet	Diet x sampling
C18:2n-6	0	16.40 ± 1.3	17.00 ± 1.5^d^	16.25 ± 2.1^d^	0.27	0.07
	30	19.00 ± 1.3	17.00 ± 0.5^d^	19.66 ± 1.6^d^	0.49	
	60	20.03 ± 2.1^a^	17.24 ± 1.4^bd^	14.60 ± 2.2^ce^	0.01	
	90	18.28 ± 2.1^a^	16.00 ± 3.9^be^	14.00 ± 2.1^ce^	0.02	
	*P* value	0.32	0.02	0.03		
C18:3n-6	0	2.06 ± 0.2	1.78 ± 0.1	1.90 ± 0.2	0.54	0.22
	30	2.59 ± 0.2	1.80 ± 0.2	2.00 ± 0.1	0.12	
	60	2.00 ± 0.2	1.30 ± 0.2	1.70 ± 0.2	0.60	
	90	2.50 ± 0.1	2.00 ± 0.3	2.00 ± 0.1	0.39	
	*P* value	0.13	0.21	0.10		
C18:3n-3	0	3.38 ± 0.7	3.85 ± 0.6^d^	2.95 ± 0.5^d^	0.09	0.08
	30	2.76 ± 0.6^b^	4.10 ± 0.3^ad^	3.32 ± 0.3^ad^	0.05	
	60	3.99 ± 0.8^b^	4.99 ± 0.8^ae^	5.56 ± 0.6^ae^	0.01	
	90	3.55 ± 1.0^c^	6.34 ± 0.8^be^	7.41 ± 1.7^ae^	0.02	
	*P* value	0.06	0.02	0.01		
C20:4n-6	0	1.65 ± 0.1	2.82 ± 0.5	1.68 ± 0.3	0.19	0.21
	30	1.58 ± 0.3	1.62 ± 0.2	1.43 ± 0.2	0.90	
	60	3.31 ± 0.7	3.00 ± 0.7	3.06 ± 0.3	0.32	
	90	2.68 ± 0.6	2.57 ± 0.7	2.12 ± 0.7	0.33	
	*P* value	0.40	0.23	0.33		
C20:5n-3	0	2.85 ± 1.0	3.20 ± 0.2	3.01 ± 1.0	0.47	0.16
	30	3.75 ± 0.4	2.90 ± 0.4	2.64 ± 0.2	0.13	
	60	3.03 ± 0.4	3.49 ± 0.6	3.73 ± 0.8	0.25	
	90	2.54 ± 0.4	3.24 ± 0.6	2.63 ± 0.5	0.63	
	*P* value	0.72	0.81	0.63		
C22:5n-3	0	2.58 ± 0.5^d^	2.71 ± 0.4^d^	2.53 ± 0.3^d^	0.34	
	30	2.17 ± 0.1^d^	2.41 ± 0.1^d^	1.94 ± 0.4^d^	0.97	0.02
	60	3.12 ± 2.0^cd^	4.58 ± 0.3^bd^	5.26 ± 0.3^ad^	0.02	
	90	5.55 ± 0.1^ce^	7.81 ± 3.6^be^	9.04 ± 2.2^ae^	0.01	
	*P* value	0.04	0.04	0.02		
C22:6n-3	0	2.28 ± 0.6	2.62 ± 0.5	2.14 ± 0.5	0.80	0.32
	30	1.96 ± 0.3	1.14 ± 0.2	0.97 ± 0.1	0.30	
	60	2.13 ± 0.7	1.33 ± 0.2	1.71 ± 0.3	0.50	
	90	1.16 ± 0.2	1.50 ± 0.1	1.28 ± 0.1	0.91	
	*P* value	0.219	0.532	0.365		
Total fatty acid (μg/mL)	0	956.65 ± 16.0	993.45 ± 28.6	918.12 ± 22.0	0.33	0.23
	30	962.56 ± 37.3	1410.22 ± 27.2	1246.04 ± 41.7	0.16	
	60	960.45 + 45.0	837.70 ± 36.9	1359.23 ± 34.3	0.20	
	90	1334.5 ± 45.0	1071.88 ± 76.2	1436.67 ± 50.0	0.67	
	*P* value	0.20	0.45	0.32		

^a, b c^means having different superscript along the same row are significantly different (*P* < 0.05). ^d, e, f^means having different superscript along the same column are significantly different (*P* < 0.05). ^g^80 % canola oil and 20 % palm oil blend

The sums and ratios of serum FA is shown in Table 5. The total saturated FA and total monounsaturated FA were not influenced by sampling time and diet. Regardless of diet, total PUFA increased (*P* < 0.05) as sampling day progressed. No significant effect of diet was observed on total PUFA on day 0, 30 and 60. However, goats fed 4 and 8 % CPOB had higher (*P* < 0.05) total PUFA than the control goats on day 90. Diet did not affect total n-3 and n-6 FA and n-6/n-3 on day 0 and 30. The control goats had higher (*P* < 0.05) total n-6 FA and n-6/n-3 and lower (*P* < 0.05) total n-3 FA compared to the oil-fed goats on day 60 and 90.

**Table 5:** Effect of diet and sampling time on sums and ratios (mean ± standard error) of fatty acids in serum of goats

		Level of dietary CPOB^g^			*P* value	
Parameter	Sampling day	0	4	8	Diet	Diet x sampling day
∑SFA	0	39.93 ± 1.4	40.18 ± 2.0	42.62 ± 1.4	0.66	0.23
	30	42.27 ± 1.2	43.08 ± 3.6	41.15 ± 2.7	0.88	
	60	43.69 ± 2.3	41.06 ± 2.4	40.43 ± 1.4	0.70	
	90	40.16 ± 2.3	38.73 ± 4.3	40.01 ± 1.8	0.16	
	*P* value	0.75	0.23	0.64		
∑MUFA	0	29.15 ± 2.4	25.74 ± 1.1	27.02 ± 2.0	0.19	0.08
	30	23.99 ± 0.7	25.68 ± 1.6	24.87 ± 1.7	0.23	
	60	19.70 ± 2.1	22.41 ± 1.3	27.11 ± 2.2	0.70	
	90	23.64 ± 4.3	21.75 ± 2.3	20.82 ± 1.8	0.45	
	*P* value	0.07	0.21	0.06		
∑PUFA	0	31.20 ± 1.4^d^	30.23 ± 2.0^d^	30.46 ± 2.3^d^	0.56	0.19
	30	33.81 ± 1.2^d^	30.23 ± 3.6^d^	32.78 ± 4.1^d^	0.55	
	60	36.64 ± 2.6^e^	36.10 ± 3.4^e^	35.6 ± 2.2^e^	0.11	
	90	36.26 ± 3.2^ae^	39.46 ± 2.5^be^	38.48 ± 3.1^be^	0.03	
	*P* value	0.04	0.02	0.03		
∑n-3	0	11.09 ± 2.4	12.38 ± 1.6^d^	10.63 ± 2.0^d^	0.82	0.04
	30	10.64 ± 1.1	10.55 ± 1.1^d^	8.87 ± 0.9^d^	0.24	
	60	11.30 ± 2.4^a^	14.56 ± 3.0^be^	16.26 ± 2.1^be^	0.03	
	90	12.80 ± 3.0^a^	18.89 ± 4.1^ae^	20.36 ± 2.2^ae^	0.01	
	*P* value	0.51	0.02	0.01		
∑n-6	0	20.11 ± 0.9^d^	21.60 ± 1.2	19.83 ± 2.2^e^	0.10	0.56
	30	23.17 ± 1.4^d^	21.61 ± 2.6	23.89 ± 2.1^e^	0.38	
	60	25.34 ± 2.0^e^	21.54 ± 1.2	19.36 ± 1.5^e^	0.91	
	90	23.46 ± 1.7^d^	20.57 ± 2.0	18.12 ± 1.3^d^	0.56	
	*P* value	0.04	0.55	0.03		
n-6:n-3	0	1.81 ± 0.3	1.94 ± 0.2^d^	1.86 ± 0.2^d^	0.22	0.03
	30	2.17 ± 0.3	1.86 ± 0.4^d^	2.70 ± 0.4^d^	0.27	
	60	2.20 ± 0.1^a^	1.74 ± 0.3^bd^	1.00 ± 0.3^ce^	0.04	
	90	1.83 ± 0.2^a^	1.49 ± 0.3^be^	0.89 ± 0.1^ce^	0.01	
	*P* value	0.18	0.02	0.01		

^a, b c^means having different superscript along the same row are significantly different (*P* < 0.05). ^d, e, f^means having different superscript along the same column are significantly different (*P* < 0.05). ^g^80 % canola oil and 20 % palm oil blend. ∑SFA = (C14:0 + C15:0 + C16:0 + C18:0), ∑MUFA = (C16:1 + C18:1), ∑PUFA = (∑n-3 + ∑n-6), ∑n-3 = (C18:3n-3 + C20:5n-3 + C22:5n-3 + C22:6n-3), ∑n-6 = (C18:2n-6+ C18:3n-6) + C20:4n-6) n-6:n-3 = (C18:2n-6 + C18:3n-6 + C20:4n-6) ÷ (C18:3n-3 + C20:5n-3 + C22:5n-3 + C22:6n-3)

### Serum antioxidants and lipid oxidation

Table 6 shows the serum TBARS value, tocopherol, total carotenoid and antioxidant enzyme activities in goats. No significant effect of sampling time, diet and interaction between sampling time and diet was observed for serum TBARS value. Neither diet nor sampling time influenced catalase, superoxide dismutase, and glutathione peroxidase activities. The serum α and γ tocopherol increased (*P* < 0.05) as the level of CPOB increased in diet on day 30, 60 and 90. Diet did not affect the concentration of total carotenoid and δ-tocopherol. Sampling time was a significant source of variation affecting serum α, γ and δ-tocopherol and total carotenoid in goats fed 4 and 8 % CPOB.

**Table 6:** Effect of diet and sampling time on serum antioxidants and lipid oxidation (mean ± standard error) in goats

Parameter	Sampling day	Level of CPOB^g^ (%)			*P* value	
		0	4	8	Diet	Diet x sampling day
TBARS (mg MDA/kg)	0	0.39 ± 0.1	0.37 ± 0.1	0.37 ± 0.1	0.57	0.55
	30	0.42 ± 0.0	0.35 ± 0.1	0.28 ± 0.1	0.35	
	60	0.35 ± 0.1	0.25 ± 0.1	0.30 ± 0.1	0.53	
	90	0.51 ± 0.1	0.39 ± 0.1	0.34 ± 0.1	0.07	
	*P* value	0.38	0.48	0.92	0.78	
Glutathione peroxidase^h^	0	57.79 ± 2.3	51.80 ± 1.9	52.90 ± 2.2	0.17	0.78
	30	62.14 ± 2.4	57.00 ± 3.2	60.11 ± 1.9	0.44	
	60	70.26 ± 2.2	54.22 ± 2.3	61.28 ± 4.2	0.76	
	90	68.20 ± 3.3	60.56 ± 3.1	58.22 ± 2.2	0.27	
	*P* value	0.14	0.76	0.67	0.12	
Catalase^i^	0	1500.94 ± 23.4	1450.75 ± 19.24	1543.43 ± 32.3	0.22	0.34
	30	1624.22 ± 27.3	1623.88 ± 28.2	1600.45 ± 33.7	0.18	
	60	1550.45 ± 22.1	1601.97 ± 41.4	1673.22 ± 23.5	0.67	
	90	1724.45 ± 23.8	1555.29 ± 26.6	1500.78 ± 30.0	0.55	
	*P* value	0.39	0.98	0.43		
Superoxide dismutase^j^	0	2.21 ± 0.1	2.30 ± 0.1	2.21 ± 0.1	0.66	0.19
	30	2.40 ± 0.2	2.37 ± 0.2	2.54 ± 0.2	0.54	
	60	2.33 ± 0.1	2.34 ± 0.1	2.34 ± 0.1	0.12	
	90	2.43 ± 0.2	2.54 ± 0.2	2.40 ± 0.1	0.32	
	*P* value	0.24	0.45	0.75		
α-tocopherol (μg/mL)	0	3.00 ± 0.3	3.20 ± 0.2^d^	2.90 ± 0.1^d^	0.63	0.25
	30	3.20 ± 0.2^c^	3.83 ± 0.2^bd^	4.22 ± 0.2^ae^	0.04	
	60	3.10 ± 0.2^c^	4.10 ± 0.1^ae^	4.67 ± 0.2^ae^	0.03	
	90	3.24 ± 0.3^b^	4.35 ± 0.3^ae^	4.80 ± 0.1^ae^	0.02	
	*P* value	0.10	0.02	0.03		
γ-tocopherol (μg/mL)	0	0.60 ± 0.1	0.58 ± 0.1^d^	0.57 ± 0.1^d^	0.34	0.22
	30	0.62 ± 0.2	0.68 ± 0.1^e^	0.72 ± 0.1^e^	0.54	
	60	0.62 ± 0.2^a^	0.70 ± 0.1^be^	0.75 ± 0.2^be^	0.01	
	90	0.64 ± 0.2^c^	0.73 ± 0.2^be^	0.80 ± 0.3^ae^	0.02	
	*P* value	0.32	0.04	0.04		
δ-tocopherol (μg/mL)	0	0.03 ± 0.0	0.03 ± 0.0^d^	0.02^d^ ± 0.0	0.62	0.58
	30	0.04 ± 0.0	0.06 ± 0.0^e^	0.06^e^ ± 0.0	0.50	
	60	0.05 ± 0.0	0.05 ± 0.0^e^	0.06^e^ ± 0.0	0.20	
	90	0.04 ± 0.0	0.06 ± 0.0^e^	0.07^e^ ± 0.0	0.11	
	*P* value	0.11	0.02	0.01		
Total carotenoid (μg /mL)	0	0.20 ± 0.0	0.20 ± 0.0^d^	0.19 ± 0.0^d^	0.43	0.27
	30	0.20 ± 0.0	0.23 ± 0.0^e^	0.25 ± 0.0^e^	0.22	
	60	0.21 ± 0.0	0.23 ± 0.0^e^	0.25 ± 0.0^e^	0.45	
	90	0.21 ± 0.0	0.25 ± 0.0^e^	0.26 ± 0.0^e^	0.23	
	*P* value	0.77	0.04	0.03		

^a, b c^means having different superscript along the same row are significantly different (*P* < 0.05). ^d, e, f^means having different superscript along the same column are significantly different (*P* < 0.05). ^g^80 % canola oil and 20 % palm oil blend. ^h^expressed as nmoles NADPH oxidized /min/mg protein. ^i^expressed as nmol.H_2_O_2_/min/mg protein. ^j^expressed as Unit/ 50 % mg protein

## Discussion

Diet is one of the prominent factors affecting serum biochemical indices in ruminants [22]. Dietary supplementation of CPOB increased serum total cholesterol and HDL cholesterol. This observation could be attributed to the increase in intestinal sterolgenesis which suggests the need to transport large amount of fat [23]. The current findings are similar to those of Bu et al. [24]. In contrast, Ponnampalam et al. [25] observed a reduction in serum total cholesterol and HDL cholesterol in lambs fed fish oil and fish meal compared to those fed basal diet. However, the authors observed similar LDL cholesterol and triglycerides between the treatments which are in tandem with the present findings.

Dietary CPOB had no effect on serum triglycerides, LDL cholesterol and VLDL cholesterol. These observations are akin to those of Li et al. [26] who observed a non-significant difference in triglycerides, VLDL cholesterol and LDL cholesterol in lactating goats fed linseed oil or soybean oil compared with those fed the control diet. Contrarily, Roy et al. [22] reported a significant increase in triglyceride levels in goats fed 4.5 % sunflower oil or soybean oil compared to those fed the control diet.

Dietary CPOB had no effect on serum glucose and total protein. This finding presumably reflects the similarity in the protein and energy contents of the diets. The similarity in serum glucose observed in the current study is in tandem with the reports of Dai et al. [27] and Roy et al. [22]. Nonetheless, Li et al. [26] reported increased serum glucose but a non-significant difference in total serum protein in lactating goats fed linseed oil or soybean oil compared with those fed the control diet.

Dietary fats can influence the fatty acid profile of serum which is an important medium for transporting fatty acids to target tissues [28]. The serum FA profile observed in the current study partly reflects the in vivo [13] and in vitro [12] ruminal fatty acids. Dietary CPOB depressed the concentration of C15:0 in the serum. The C15:0 is an odd chain FA obtained solely from rumen microbial biomass [29]. Thus, the decrease in its proportion with oil supplementation could be due to the effect of unprotected CPOB on rumen microbial ecology and metabolism which reduced the concentration of odd chain fatty acid in microbial biomass [13, 29, 30] or the flow of microbial biomass to the duodenum [28].

Dietary CPOB had no effect on the concentration of C14:0 and C16:0. Corroborating the present observation, Karami et al. [8] did not observe significant difference in the proportion of serum C14:0 and C16:0 between goats fed 3 % canola oil and those fed 3 % palm oil. In contrast, supplementation of 3.3 % canola oil, canolamide or blend of canola oil and canolamide reduced serum C14:0 and C16:0 in dairy cows [31]. The concentration of C18:0 was unaffected by oil supplementation. This observation is in line with that of Chang et al. [32].

The increase in serum C18:1n-9 in goats fed 4 and 8 % CPOB compared to the control diet on day 60 presumably reflect dietary intake of C18:1n-9. This observation could also be due to the desaturation of the absorbed C18:0 by tissue desaturase. This finding concurs with those of Loor et al. [31] who observed that dairy cattle fed 3.3 % canola oil, canolamide or blend of canola oil and canolamide had higher serum C18:1n-9 compared with those fed control diet. In addition, Ahmadi sheik et al. [33] observed that lambs fed extruded canola and cotton seeds had higher plasma C18:1n-9 compared to those fed control diets.

The reduction of serum C18:2n-6 in goats fed 4 and 8 % CPOB relative to those fed the control diet could be due to its lower dietary (Table 1) and ruminal concentrations [13]. It could also be due to the increase in serum long chain n-3 fatty acids (e.g., C22:5n-3) which preferentially substituted for C18:2n-6.

Dietary CPOB enhanced the concentration of serum C18:3n-3 in goats. This observation reflects the FA composition of the dietary treatments suggesting that some C18:3n-3 escaped ruminal biohydrogenation. Companion in vitro [12] and in vivo [13] studies showed that the ruminal concentration of C18:3n-3 increased as the level of CPOB increased in the diet. The current observation is in tandem with those of Karami et al. [8] who observed a significant increase in the plasma C18:3n-3 of goats fed 3 % canola oil compared to those fed 3 % palm oil.

The increase in serum C22:5n-3 in goats fed 4 and 8 % CPOB on day 60 and 90 could be due to the increase in the proportion of C18:3n-3 suggesting considerable in vivo elongation of C18:3n-3. This finding is in tandem with those of Goodridge et al. [34] and Kim et al. [35] who observed significant increase in serum long chain n-3 FA in cattle fed flax seed compared to those fed the control diet. Contrarily, Karami et al. [8] did not observed significant differences in the proportion of long chain n-3 FA between goats fed 3 % canola oil versus 3 % palm despite the increase in C18:3n-3 in the plasma of goats fed canola oil.

Dietary CPOB did not affect serum total saturated fatty acid. Sampling time was a significant source of variation influencing the total MUFA of goats fed control diet and 8 % CPOB; however the changes were inconsistent. The significant decrease in the n-6/n-3 in goats fed 4 and 8 % CPOB on day 60 and 90 could be attributed to the higher total n-3 FA observed in the serum of these animals.

Oxidative stress in tissues is caused by the imbalance between generation of free radicals and antioxidant defense systems [3–7]. Increase in tissue unsaturated fatty acids in the presence of a weak antioxidant defense system could trigger lipid oxidation [3–6]. Given the increase in serum n-3 FA in goats fed 4 and 8 % CPOB on day 60 and 90, one would expect an increase in serum TBARS. Thus, the similarity in TBARS value across the treatments especially on day 60 and 90 may be due to the increase in α and γ-tocopherol observed in serum of goats fed 4 and 8 % CPOB compared to those fed control diet. This is particularly true for antioxidant enzymes whose activities have been reported to increase with increase in oxidative stress [5, 36]. Increased glutathione peroxidase [37], superoxide dismutase [38] and catalase [39] activities in response to dietary unsaturated fatty acids have been documented. In contrast, dietary oxidized fish oil depressed plasma catalase, superoxide dismutase and glutathione peroxidase activities in pigs [6]. The current findings indicate that the significant increase in the serum α and γ tocopherol compensated well for the increase in the n-3 fatty acids. This finding corroborates the report of Karami et al. [8] who observed that goats fed 3 % canola oil had similar plasma TBARS value as those fed 3 % palm oil throughout the feeding trial.

The significant increase in the concentration of α and γ tocopherol in goats fed 4 and 8 % CPOB relative to the control diet presumably reflect the antioxidant contents of the dietary treatments (Table 1). Tocopherol is a fat soluble vitamin [40]. Thus, the increase in fat content of the diet as dietary CPOB increased might have aided the absorption and deposition of these antioxidants in the serum of oil-fed goats compared to the control goats. The current observation is akin to the findings of Soler-Velasquez et al. [41] who observed that swine fed 5 and 10 % canola oil had higher serum α-tocopherol compared with those fed control diet. Similarly, Jakobsen et al. [42] observed that increasing the concentration of α, γ and δ-tocopherol in chicken’s diet increased the blood plasma contents of the tocopherol. The increase in α, γ and δ-tocopherol and total carotenoid in serum of oil-fed goats as sampling day progressed reflects dietary antioxidant contents resulting from the palm oil and canola oil in the diet.

## Conclusion

The results of the current study demonstrate that dietary CPOB enhanced the proportion of serum n-3 fatty acids without compromising lipid oxidative stability and biochemical parameters in goats.

## Abbreviations

CPOB: 80 % canola oil and 20 % palm oil blend.
FA: fatty acid
GPx: glutathione peroxidase
HDL: high density lipoprotein
LDL: low density lipoprotein
MUFA: monounsaturated fatty acid
PUFA: polyunsaturated fatty acid
SFA: saturated fatty acid
TBARS: thiobarbituric reactive substance
VLDL: very low density lipoprotein
